# Glucocorticoid Treatment Dampens Kynurenine Pathway Activation and Cytokine Release in Immune Stimulated Human Microglia

**DOI:** 10.1177/11786469261416782

**Published:** 2026-01-23

**Authors:** Martina Esposito Soccoio, Robert P. Mason, Julien Devilliers, Roberto Feuda, Mary E. W. Collier, Flaviano Giorgini

**Affiliations:** 1 Division of Genetics and Genome Biology, University of Leicester, UK

**Keywords:** microglia, kynurenine pathway, kynurenine 3-monooxygenase, inflammatory cytokines, dexamethasone

## Abstract

As a first line of defence for the central nervous system (CNS), microglia play a critical role in maintaining homeostasis within the brain. Upon detection of damage or threats, activated cells release factors to communicate and potentiate immune responses. This activation also increases activity of the kynurenine pathway (KP) and alters expression of key KP enzymes such as indoleamine 2,3-dioxygenase (IDO-1) and kynurenine 3-monooxygenase (KMO), both major contributors in the pathology of several neurodegenerative and psychiatric disorders. This study investigated the impact of pro-inflammatory stimuli on the C20 human microglial cell line, focussing on the regulation of *KMO* and *IDO-1* expression, and the production of cytokines. Additionally, we explored whether the anti-inflammatory effects of dexamethasone (DEXA) influenced these outcomes. This additional characterisation of a physiologically relevant human microglial cell line offers a novel and reliable platform for investigating human-specific microglial biology and function. C20 were challenged for 24 hours with cytokines or lipopolysaccharide (LPS). Gene expression was measured by RT-qPCR and excreted cytokines were quantified using a multiplex array. Our results showed up-regulation of *IDO-1* and *KMO* transcripts, and increased release of pro- and anti-inflammatory cytokines. Notably, these effects were significantly dampened by pre-incubation with DEXA. Furthermore, transcriptomic analyses supported these data by highlighting TNF-α-activated enriched pathways, as well as those down-regulated in samples co-treated with DEXA. This study contributes to the understanding of key mechanisms regulated in human microglia by immune challenges and supports the crucial role of synthetic glucocorticoids (GCs) in moderating the microglial immune response induced by pro-inflammatory signals. These data support the use of GCs as possible therapeutic interventions for diseases associated with neuroinflammation, particularly those with altered KP metabolism.

## Introduction

Originating from precursors of the embryonic yolk sac that move to the developing brain during embryogenesis,^1^ microglia are ontogenically classified as immune cells and thus the first line of defence for the central nervous system (CNS). The primary function of microglia is to maintain homeostasis within the CNS, therefore in physiological conditions, they can be typically found in ‘surveillance mode’, which reflects the unique ability of these cells to extend and retract their fine processes in order to continuously inspect the brain parenchyma.^2^ This characteristic becomes particularly important when microglia need to promptly respond to signals coming from the extracellular environment that could affect brain homeostasis. Microglial cells have direct interactions with neurons and thereby influence synaptic plasticity through the release of specialised messengers such as tumour necrosis factor alpha (TNF-α) or interleukin 1 beta (IL-1β).^3,4^ These cytokines can regulate synaptic transmission by interacting with receptors present on the neuronal plasma membrane^3,5^ or by modulating their activity through phosphorylation.^4^ In addition, microglia produce neurotrophic factors (eg, basic fibroblast growth factor, nerve growth factor) which stimulate cellular differentiation and promote the genesis of neuronal circuitry during early development as well as enhancing survival and maintenance in adulthood,^6^ highlighting their vital role in the dynamics of the CNS.

In case of damage or potential threats to the CNS, a series of morphological and phenotypic changes mark the transition of microglial cells into a different status commonly known as ‘activation’^7^. The set of receptors expressed on the plasma membrane form the so-called microglia ‘sensome’ which is responsible for perceiving alterations in the surrounding environment including changes in pH, the presence of pathogens or misfolded proteins, and can thus initiate an accurate cellular response.^8^ Accordingly, activated microglia start to release various factors such as chemokines, cytokines and other inflammatory signals to communicate the presence of damage or inflammation to other cells, as well as to potentiate the immune response.^9^ Among these, the release of interferon-gamma (IFN-γ) triggers an increase in the expression of the enzyme indoleamine 2,3-dioxygenase-1 (IDO-1)^10^ which is responsible for the initiation of the kynurenine pathway (KP) in the brain.

**Figure 1.**
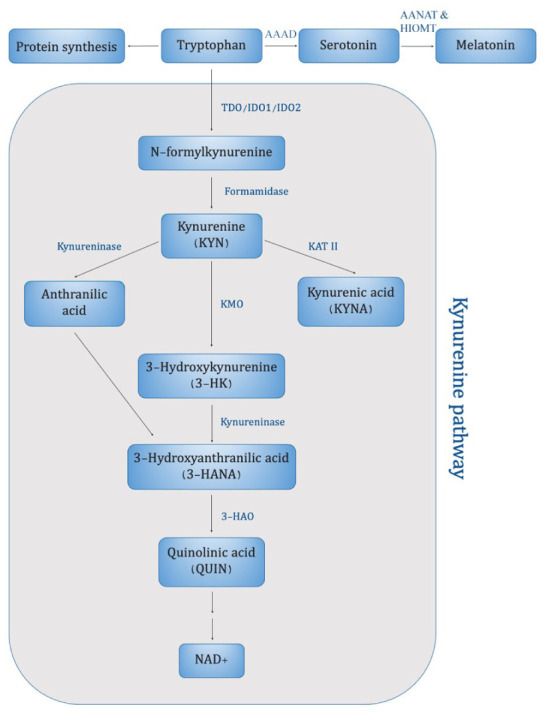
Simplified scheme of the KP in mammals. The KP is initiated when TRP is converted to N-formylkynurenine by TDO, IDO1 or IDO2. The following reaction determines the production of KYN which can be transformed to anthranilic acid by kynureninase, into 3-HK by KMO or into KYNA by KAT II. From 3-HK the pathway continues until the formation of QUIN and ultimately NAD^+^. Enzymes are shown in blue. Abbreviation: AAAD, aromatic l-amino acid decarboxylase; AANAT, serotonin-N-acetyltransferase; HIOMT, hydroxyindole-O-methyltransferase; TDO, tryptophan 2,3-dioxygenase; IDO1 and 2, indoleamine 2,3-dioxygenase 1 and 2; KMO, kynurenine 3-monooxygenase; KAT II, kynurenine aminotransferase II; 3-HAO, 3-hydroxyanthranilate 3,4-dioxygenase.

In mammals, more than 95% of free tryptophan (TRP) is normally degraded in the liver through the KP by the action of the enzyme tryptophan 2,3-dioxygenase (TDO)^11,12^ (Figure 1). However, the initiation of an immune response leads to an additional mode of TRP catabolism from extrahepatic routes such as within the brain, which is regulated by IDO-1. Both TDO and IDO-1 convert TRP into N′-formylkynurenine^13^ which in turn is metabolised into L-kynurenine (L-KYN) by the enzyme formamidase.^14^ L-KYN synthesis represents a key step in the KP as it marks the origin of 2 independent branches of this metabolic cascade in the CNS. Indeed, astrocytes and microglia express a unique set of downstream KP enzymes producing different neuroactive metabolites commonly known as kynurenines.^15^ More specifically, astrocytes possess the enzyme kynurenine aminotransferase II (KAT-II), which converts L-KYN into kynurenic acid (KYNA).^16^ Inside microglial cells, L-KYN is the substrate of kynurenine 3-monooxygenase (KMO), which converts L-KYN into 3-hydroxykynurenine (3-HK).^17^ The latter then undergoes a series of reactions that lead to the production of quinolinic acid (QUIN). Once synthesised by the respective glial cells, KYNA and QUIN are promptly released into the extracellular milieu acting on the pre- and postsynaptic receptors localised on the membrane of glutamatergic neurons.

It has been shown that the KP can be activated by immune stimulation.^15,18,19^ *KMO* expression has been reported to be significantly increased by lipopolysaccharide (LPS) stimulation in microglia both in vivo^20^ and in vitro.^18,21^ IL-1β stimulation has also been found to upregulate *KMO* transcript levels in human hippocampal progenitor cells.^22^ In addition, Liu et al^23^ described a similar effect on *KMO* expression in pancreatic rat islets cells following treatment with IL-1β. Under neuroinflammatory conditions, the upregulation of *KMO* expression and activity by inflammatory mediators causes a shift of L-KYN metabolism away from KYNA towards the production of 3-HK and QUIN.^24^ Unceasing activation of N-methyl D-aspartate (NMDA) receptors by QUIN leads to excitotoxicity, a phenomenon by which neuronal injury or death is induced by excessive cellular stimulation through neurotransmitters, in this case, glutamate.^25^ In neurons, this generates a series of detrimental events such as oxidative stress, mitochondrial dysfunction and the activation of proteases, but it also initiates a degenerative chain in the brain that spreads across to the neighbouring cells.^26^ Indeed, a shared feature across various neurodegenerative diseases is the shift of the KP flux towards the QUIN producing arm of the pathway; where the concentration of 3-HK and QUIN are increased in the brain, accompanied by a reduction of the levels of KYNA.^27^

To ensure homeostasis within the CNS, any immunological response needs to be balanced by a reciprocal anti-inflammatory action to promote tissue repair, regeneration and remapping of neural networks. Chronic inflammation is recognised as a major factor in neurodegeneration, and it has been shown that microglia are activated in almost all neurological disorders^28^ exhibiting a pro-inflammatory phenotype, which may exacerbate the pathological condition consequent to the persistent release of multiple cytotoxic factors.^29^ Inflammation within the CNS, generally associated with a dysregulated immune response, is not only a major cause of neurodegenerative diseases, but also has a critical impact on mental health.^30^ In particular, alterations related to microglia activation have been linked to the pathology of major depressive disorder^31^ and schizophrenia.^32^ Similarly, dysfunction of the kynurenine pathway has been found to have a critical impact on the development and symptomatology of several mental health disorders.^33^ Thus, a strategy focussed on understanding microglial function in association with approaches aimed at modulating neuroinflammation might lead to beneficial treatments for several diseases affecting the CNS. To date, most insights into microglial activation have been derived from in vivo rodent studies or in vitro approaches employing primary cultures or transformed cell lines from various mammalian species.^34^ While these models have been invaluable, primary microglial cultures are technically challenging and costly whilst rodent microglia might exhibit pronounced species-specific differences from their human counterparts.^34^ A physiologically relevant human microglial cell line fills a critical gap, providing practical advantages in terms of scalability and reproducibility in combination with enabling direct investigation of human-specific microglial biology.

The aim of this study was to explore possible changes occurring in the C20 human microglial cell line following different immune stimulations with a specific focus on the release of pro-inflammatory biomarkers as indicators of microglia activation, as well as the expression of key enzymes involved in the KP. Furthermore, the use of dexamethasone (DEXA), a synthetic glucocorticoid (GC) with anti-inflammatory properties,^35-37^ was also implemented to test the impact that this intervention has on microglial cellular response to inflammatory cytokines. Finally, the use of RNA-seq in combination with Gene Ontology (GO)-term enrichment analysis was employed to compare the transcriptomic profiles of cells treated with TNF-α alone or in combination with DEXA and establish enrichment of molecular pathways connected to microglial immune response and its modulation through TNF-α. In doing so, we aimed to establish and provide an additional characterisation of a robust, physiologically relevant human microglial cell line, offering a novel and accessible platform for investigating human-specific microglial biology and function.

We found that treatment of C20 cells with several pro-inflammatory stimuli activated the KP through the up-regulation of both *IDO-1* and *KMO* gene expression. Similarly, the treatment with IL-1β or TNF-α caused an increase in the release of several pro- and anti-inflammatory cytokines from microglial cells. Notably, the addition of DEXA was sufficient to reduce the levels of KP enzyme transcripts as well as the cytokines released in the media in response to pro-inflammatory cytokines, highlighting the possible utility of GCs in conditions of neuroinflammation generated by over-activated microglia. Furthermore, analysis of the transcriptome also supported the data obtained by highlighting enriched pathways activated by exposure to TNF-α and those downregulated in samples pre-treated with DEXA.

## Materials and Methods

### Cell Culture and Stimulation of Cells With Cytokines

The C20 human microglial cell line was obtained from David Alvarez-Carbonell (Case Western Reserve University) and was originally generated by Garcia-Mesa et al.^38^ C20 cells were cultured in Dulbecco’s modified Eagle medium high glucose (DMEM GlutaMAX™; Thermo Fisher Scientific, Loughborough, UK) supplemented with 10% foetal bovine serum (FBS; Thermo Fisher Scientific), penicillin (100 units/ml) and streptomycin (100 μg/ml). Cells were incubated at 37 °C under 5% CO_2_ and were passaged at 80% to 90% confluency. Cells were used until passage 25 was reached. Cells were seeded onto plates in complete DMEM and were incubated at 37 °C overnight. The following day, the medium was aspirated, cells were washed twice with PBS and fresh Macrophage Serum Free Medium (MSFM; Gibco/Thermo Fisher Scientific) was added. After 24 hours incubation at 37 °C, the following human recombinant pro-inflammatory cytokines were added: IL-1β (20 ng/mL; Peprotech, Rocky Hill, NJ, USA), TNF-α (50 ng/mL; Peprotech) or IFN-γ (20 ng/mL; Peprotech). These molecules were chosen as they are the main microglial endogenous stimulants in the brain. Alternatively, cells were treated with LPS (1 μg/ml; Associates of CAPE COD, Inc, MA, USA). Pre-treatment with DEXA (1 μM; Santa Cruz Biotechnology, TX, USA) in dimethyl sulfoxide (DMSO) was carried out for 1 hour prior to immune stimulation. DEXA cytotoxicity was excluded by using the WST-8 kit (Abcam, MA, USA) measuring cell absorbance at 460 nm.

### RNA Isolation and qPCR for the Quantification of KP Enzyme Expression

C20 cells were seeded at a density of 62 500 cells/well in 6-well plates in DMEM and incubated for 24 hours at 37 °C. The following day the medium was aspirated, cells were washed with PBS and warm MSFM was added. Samples were incubated again for 24 hours at 37 °C. Subsequently, cells were challenged with the specific stimuli, whereas only medium was added to control wells. Cells were then incubated for a further 24 hours and RNA was isolated using TRIsure™ (Bioline/Meridian BioScience, London, UK)/chloroform extraction. RNA was resuspended in 30 µl of RNase-free water and cDNA was obtained by using the QuantiTect^®^ Reverse Transcriptase Kit (Qiagen, Manchester, UK). One µg of RNA sample was mixed with 2 μl of 7× gDNA wipeout buffer and RNAse free water to a final volume of 14 µl and then incubated at 42 °C for 2 minutes to eliminate any contaminating genomic DNA. cDNA was synthesised by adding the previous solution to 4 μl of 5× Quantiscript RT Buffer, 1 μl of RT primers and 1 µl of Quantiscript Reverse Transcriptase. The same mix was prepared but adding RNAse free water instead of the enzyme for the no RT negative control. Samples were incubated at 42 °C for 30 minutes followed by 3 minutes at 95 °C. qPCR was carried out as follows: 1 μl of cDNA, 2 µl of each primer (3 µM stock), 5 μl of PrecisionPLUS qPCR Master Mix (Primerdesign) and 2 μl of RNase free water. Samples were dispensed in triplicate into a 96-well plate and the run was performed in a Roche Light Cycler^®^ 480 using the following cycling conditions: (1) initial denaturation: 95 °C for 10 minutes; (2) denaturation: 95 °C for 15 seconds; (3) annealing: 60 °C for 1 minute. Steps 2 and 3 were repeated for 40 cycles. The primers (Table 1) specificity was verified by the analysis of a melting curve produced at the end of every annealing step combined with a final step of amplification. The reference gene *RPLP0* was utilised for the normalisation of the gene expression levels using the 2−ΔΔ*Ct* method.^39^

**Table 1.** List of Oligonucleotides Used for the KP Enzyme Expression in C20 Cells.

Primer name	Sequence (5′ → 3′)	Description
hRPLP0 F	GCTGCTGCCCGTGCTGGTG	Forward primer for *hRPLP0*
hRPLP0 R	TGGTGCCCCTGGAGATTTTAGTGG	Reverse primer for *hRPLP0*
hKMO F	GAATGCGGGCTTTGAAGAC	Forward primer for *hKMO*
hKMO R	ACAGGAAGACACAAACTAAGGT	Reverse primer for *hKMO*
hIDO F	AGGCAACCCCCAGCTATCAG	Forward primer for *hIDO1*
hIDO R	ATTTTCCACTCACCTCCACCAG	Reverse primer for *hIDO1*

### Quantification of Cytokines in C20 Conditioned Media

C20 cells were seeded at a density of 62 500 cells/well in quadruplicate in 6-well plates and cultured in DMEM for 24 hours at 37 °C. The following day cells were washed with PBS and fresh MSFM was added. After 24 hours, microglia were then stimulated with pro-inflammatory cytokines alone or in combination with DEXA and incubated for a further 24 hours. From each well the conditioned media was collected and centrifuged at 1000*g* for 5 minutes at 4 °C to remove cells. The supernatant was then transferred to a fresh tube and snap frozen on dry ice before being stored at −80 °C. Quantitative estimation of secreted cytokines was evaluated using the V-PLEX Proinflammatory panel 1 Human Kit (Meso Scale Discovery, Rockville, MD, USA) following the manufacturer’s guidelines. Samples were diluted 2-fold if untreated or higher if stimulated. The experimental plate consisted of a 10-multispot 96-well plate pre-coated with capture antibodies which allowed to individually measure the concentration of the following cytokines: IFN-γ, IL-1β, IL-2, IL-4, IL-6, IL-8, IL-10, IL-12p70, IL-13 and TNF-α during the same run. Each well of the plate was washed 3 times with 150 μl of wash buffer (PBS + 0.05% [v/v] Tween-20) followed by the addition of 50 μl of sample or calibrator loaded in duplicates. The plate was then sealed with adhesive foil and incubated at room temperature with shaking at 1000 rpm for 2 hours. Afterwards, the washing step was repeated as previously described and 25 μl of detection antibody solution was added per well. The plate was sealed again and incubated with shaking at 1000 rpm for another 2 hours at room temperature. Before being analysed on an MSD instrument, a last wash of the plate was performed and 150 μl of 2× reading buffer was dispensed into each well. Plates were read on a MESO QuickPlex SQ 120MM and data was analysed using the DISCOVERY WORKBENCH 4.0 Analysis Software. Standard curves for each cytokine were used to calculate the concentrations of cytokines in each sample.

### Statistical Analyses

The statistical analyses were performed using the software Prism version 8.4.3 (GraphPad, Inc, San Diego, CA). The legends for each figure describe the specific details of the performed tests. A *P* < .05 was considered significant.

### Cell Preparation and RNA Extraction for mRNA Sequencing

C20 were seeded and stimulated as previously described. The medium was then removed, and the cells were washed with PBS. RNA was extracted according to the manufacturer’s instructions. Briefly, 200 µl of 1-Thioglycerol/Homogenisation Solution mix (Promega, Southampton, UK) was added to the cells, followed by 30 seconds incubation on ice. Cells were then scraped and collected in a fresh Eppendorf tube. Next, 200 µl of lysis buffer was added and samples were vortexed for 15 seconds. Maxwell^®^ 16 LEV Cartridges (Promega) were then prepared as follows: the required number of cartridges was positioned in the deck tray. In the designated well, a plunger and an elution tube filled with 50 μl of Nuclease-Free H_2_O were inserted for each cartridge. Lysates were loaded into the first well whilst 5 µl of DNAse was loaded into the fourth well of the cartridge. Once all samples were loaded, the tray was transferred to the Maxwell instrument (Promega) and processed using the programme ‘simplyRNA Cells’ from the Maxwell software.

### RNA Quality Control and mRNA Sequencing

RNA quality and concentration were assessed using the Agilent RNA 6000 Nano kit on an Agilent 2100 Bioanalyzer System. Samples with the best combination of high concentration and RNA integrity number (>9) were chosen to be shipped to Novogene Cambridge Genomic Centre (Novogene Company Ltd, Cambridge, UK) for mRNA library preparation (poly A enrichment) and sequencing using Ilumina PE150, generating at least 40 M reads/sample.

### Bioinformatic Analysis

The quality of the reads was evaluated using FastQC 0.11.9^40^ and summarised using MultiQC 1.12.^41^ Reads were then aligned to the human reference genome (GRCh38, https://www.ncbi.nlm.nih.gov/genome/guide/human/) from NCBI, and transcripts abundance was evaluated using Kallisto 0.44.0.^42^ After filtering out transcripts with <10 counts, transcript counts were then normalised by library and condition following the DESeq2 pipeline.^42^ Differential transcript expression between the conditions was computed in the DESeq2 package version 1.36.0^43^ using R version 4.2.2 (R Core Team, 2022), and *P* values were adjusted following the Benjamini-Hochberg method.^44^ The dataset list obtained was filtered selecting genes with an adjusted *P* ⩽ .01 and a minimum of 2.0 log fold-change (logFC). The scripts used are available at: https://github.com/juliendevi/RNA-seq_human_cells.

### Analysis of DEGs Using STRING Protein-Protein Interaction Network Analysis

In order to establish potentially enriched biological pathways connecting the identified DEGs, the corresponding encoded proteins (divided into up- and down-regulated genes depending on the conditions compared) were analysed using the STRING protein-protein interaction network database.^45^ Predicted interactions were calculated based on 7 types of evidence (fusion, neighbourhood, co-occurrence, experimental, text mining, database and co-expression). A high/highest interaction score (0.7-0.9 confidence) was chosen so that only interactions above this score were included in the network.

## Results

### *KMO* and *IDO-1* mRNA Expression Are Upregulated Upon Immune Stimulation

The C20 human microglia cell line was immune stimulated with pro-inflammatory cytokines (IL-1β, TNF-α and IFN-γ) or LPS after being cultured in serum-free media for 24 hours. Compounds were added alone or in combination, and cells were again incubated for 24 hours, and the presence of the *KMO* gene transcripts were quantified by RT-qPCR.

**Figure 2.**
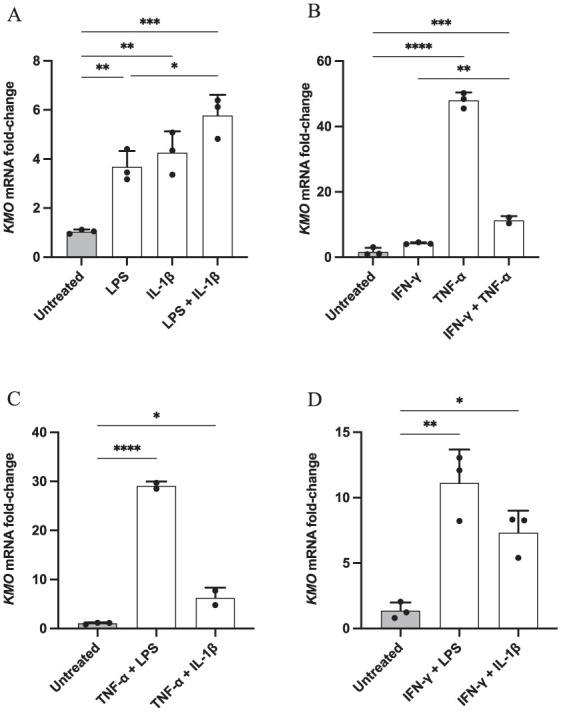
KMO expression is increased upon immune stimulation in human microglia. C20 cells were seeded in triplicates at a density of 62 500 cells/well in a 6-well plate in DMEM and incubated for 24 hours at 37 °C. Media was replaced with MSFM after 24 hours. Subsequently samples were treated with: (A) LPS (1 μg/ml), (B) TNF-α (50 ng/ml), (A) IL-1β (20 ng/ml) or (B) IFN-γ (20 ng/ml) as single or combined stimulations, and (A – D) incubated again for 24 hours. The total RNA was then extracted and gene expression was measured by qPCR. Data are presented as the fold of change versus the reference gene (RPLP0) mean ± SD, N = 3. Statistics were determined by 1-way ANOVA followed by Tukey’s multiple comparison tests. *P ⩽ .05. **P ⩽ .005. ***P ⩽ .0005. ****P ⩽ .0001.

As expected, a very low level of *KMO* expression was observed in untreated microglia (Figure 2). On the contrary, when cells were immune challenged, there was a significant up-regulation of *KMO* mRNA levels following treatment with inflammatory stimuli (Figures 2A-D), with the only exception being for samples treated with IFN-γ alone (Figure 2B). Notably, a comparison of all the different conditions revealed that treatment of cells with TNF-α alone (Figure 2B) or TNF-α combined with LPS (Figure 2C) resulted in the greatest increase in *KMO* expression with a ~48- and a ~29-fold change, respectively, in relation to the untreated control. The rest of the treatments produced an increase in *KMO* mRNA expression ranging between ~3.7- and ~11.3-fold change as supportive evidence that the KMO-branch of the KP could be increased during neuroinflammatory conditions.

**Figure 3.**
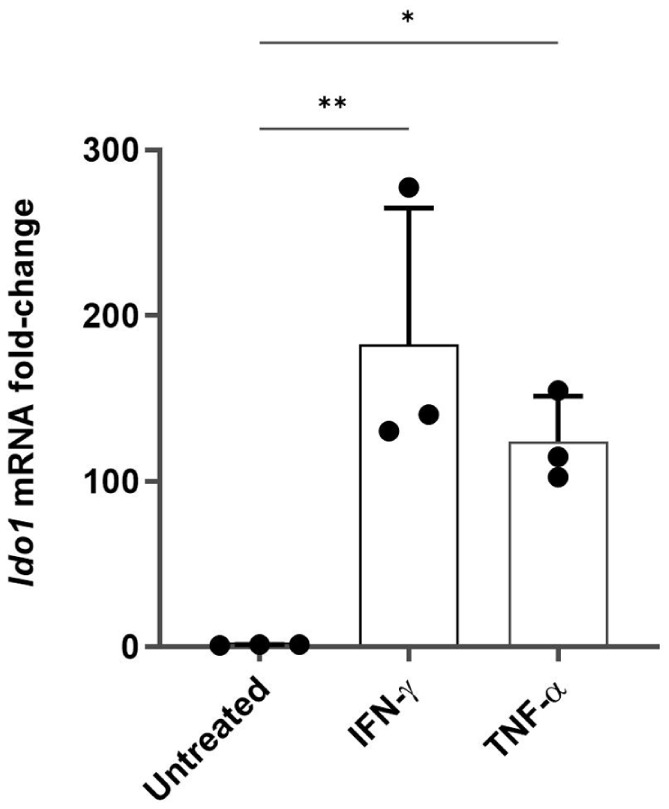
IDO-1 expression is up-regulated in immune-challenged human microglia. C20 cells were seeded at a density of 62 500 cells/well in a 6-well plate in DMEM and incubated for 24 hours at 37 °C. Media was then removed and MSFM was added. The following day cells were treated with IFN-γ (20 ng/ml) or TNF-α (50 ng/ml) and incubated again for 24 hours. The RNA was extracted and IDO-1 expression was verified by qPCR. The data are presented as the fold of change versus the reference gene (RPLP0) mean ± SD, N = 3. One-way ANOVA followed by Tukey’s multiple comparison tests. *P ⩽ .05. **P ⩽ .005.

To further characterise the KP activation following an immune challenge, the expression of *IDO-1*, the first and limiting enzyme of this catabolic route, was also quantified in microglial cells. C20 cells were immune stimulated for 24 hours with TNF-α (chosen as the most influential cytokine in *KMO* expression by the previous experiment) and IFN-γ, which was instead used as positive control, considering the recognised role that this cytokine has on *IDO-1* induction.^10^ qPCR analysis revealed that *IDO-1* mRNA expression was dramatically increased in both conditions (Figure 3); a ~135-fold increase was observed in IFN-γ-treated cells, whilst a ~33-fold change was measured in TNF-α-challenged samples compared to the untreated control, further confirming the influence that immune stimulation has on the initiation of the KP.

### Dexamethasone Administration Decreases the Expression of KP Enzymes in TNF-α and IL-1β-Challenged Microglia

**Figure 4.**
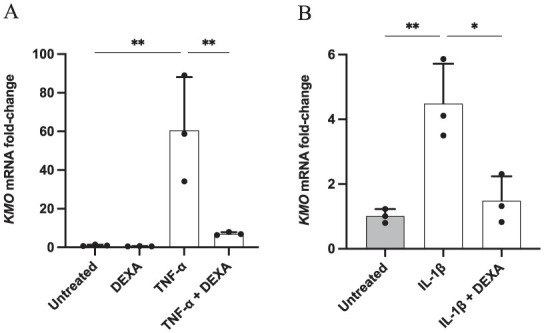
Pro-inflammatory cytokine-induced KMO expression is dampened by DEXA addition in human microglia. C20 cells were seeded at a density of 62 500 cells/well in a 6-well plate in DMEM and incubated for 24 hours at 37 °C. Media was then removed and MSFM was added. The following day cells were pre-treated with DEXA (1 μM) for 1 hour before adding (A) TNF-α (50 ng/ml) or (B) IL-1β (20 ng/ml). After 24 hours the RNA was extracted and KMO mRNA levels were measured by qPCR. Data are presented as the fold of change versus the reference gene (RPLP0) mean ± SD, N = 3. One-way ANOVA followed by Tukey’s multiple comparison tests. **P ⩽ .005. *P ⩽ .05.

The addition of the glucocorticoid DEXA (1 µM) to C20 cells was specifically combined with TNF-α and IL-1β stimulations following the already previously described protocol. qPCR measurement of *KMO* mRNA levels showed that samples co-treated with TNF-α and DEXA exhibited a ~8.5-fold down-regulation of *KMO* expression compared to cells solely exposed to TNF-α (Figure 4A). Similarly, the increased transcript levels of *KMO* due to IL-1β treatment were significantly reduced in the presence of DEXA (Figure 4B). A negative control was also performed by treating microglia with DEXA alone to assess a possible influence of DEXA on *KMO* expression. Results showed no significant difference in *KMO* mRNA expression in cells treated with DEXA compared to the untreated samples, suggesting no direct effect of DEXA on the transcription of the *KMO* gene.

**Figure 5.**
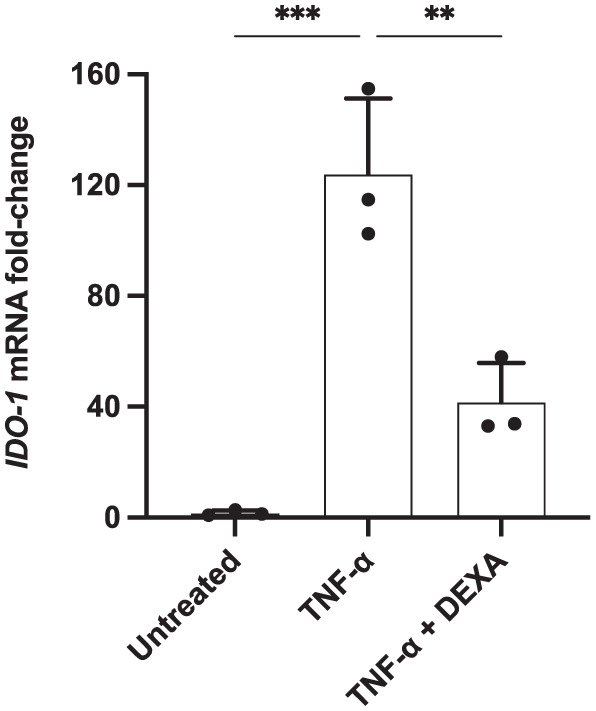
DEXA decreased IDO-1 expression in pro-inflammatory cytokine-stimulated human microglia. C20 cells were seeded at a density of 62 500 cells/well in a 6-well plate in DMEM and incubated for 24 hours at 37 °C. Media was then removed and MSFM was added. After 24 hours cells were pre-treated for 1 hour with DEXA (1 μM) and then with TNF-α (50 ng/ml). After 24 hours incubation, the RNA was extracted and IDO-1 expression was quantified by qPCR. The data are presented as the fold of change versus gene reference (RPLP0) mean ± SD, N = 3. One-way ANOVA followed by Tukey’s multiple comparison tests. ***P ⩽ .0005. **P ⩽ .005.

The impact of DEXA treatment on the KP was additionally explored by measuring *IDO-1* mRNA levels. C20 cells were hence stimulated for 24 hours with TNF-α, alone or combined with DEXA. Consistent with previous results, the activation of the KP, as a consequence of the cytokine stimulation, was reflected in the up-regulation of *IDO-1* expression, with a ~124-fold increase in TNF-α-treated samples (Figure 5). Notably, the addition of DEXA in combination with this pro-inflammatory cytokine resulted in an almost 3-fold decrease in *IDO-1* mRNA expression compared to cells treated with TNF-α alone.

### DEXA Treatment Modulates Pro- and Anti-Inflammatory Cytokine Release From Cytokine-Stimulated C20 Microglial Cells

**Figure 6.**
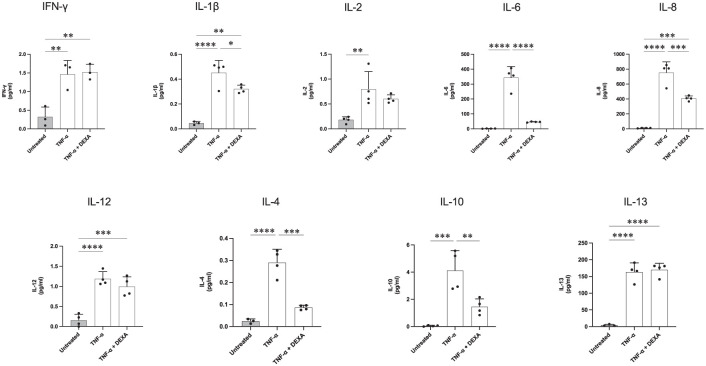
Cytokine release from TNF-α-stimulated human microglia is modulated by the addition of DEXA. C20 cells were seeded at 62 500 cells/well in quadruplicate in a 6-well plate. Complete medium was replaced with serum-free media after 24 hours. The following day cells were stimulated with TNF-α (50 ng/ml) alone or in combination with 1 hour pre-treatment with DEXA (1 μM). After 24 hours the conditioned media was collected and centrifuged for 5 minutes at 1000*g* 4 °C. On the day of the assay, media was thawed and diluted before being added to an MSD MULTI-SPOT^®^ 96-well microplate. The presence of 10 different cytokines was measured by the MSD plate reader. Data are presented as the specific cytokine concentration mean ± SD, N = 4. One-way ANOVA, Tukey’s test. ****P ⩽ .0001. ***P ⩽ .0005. **P ⩽ .005. *P ⩽ .05.

**Figure 7.**
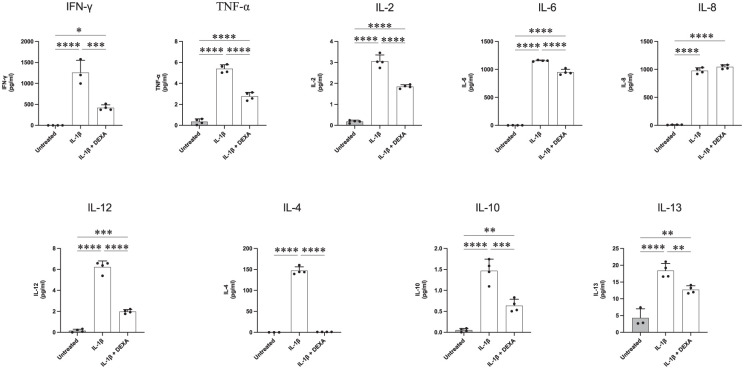
Cytokine release from IL-1β-stimulated human microglia is modulated by the addition of DEXA. C20 cells were seeded at 62 500 cells/well in quadruplicate in a 6-well plate. Complete medium was replaced with serum-free media after 24 hours. The following day cells were stimulated with IL-1β (20 ng/ml) alone or in combination with 1 hour DEXA pre-treatment (1 μM). After 24 hours the conditioned media was collected and centrifuged for 5 minutes at 1000*g* 4 °C. On the day of the assay, media was thawed and diluted before being added to an MSD MULTI-SPOT^®^ 96-well microplate. The presence of 10 different cytokines was measured by the MSD plate reader. Data are presented as the specific cytokine concentration mean ± SD, N = 4. One-way ANOVA, Tukey’s test. ****P ⩽ .0001. ***P ⩽ .0005. **P ⩽ .005. *P ⩽ .05.

Stimulation of C20 cells with IL-1β or TNF-α was performed as described above and the conditioned media of treated and untreated cells was collected to simultaneously measure the concentration of 10 different pro- and anti-inflammatory cytokines. All cytokine concentrations were lower in controls compared to immune-stimulated samples (Figures 6 and 7). This was expected considering that in physiological conditions these cytokines are not normally produced by microglia, and also indicated the inactivated status of the untreated cultured cells. On the other hand, stimulation with either TNF-α or IL-1β resulted in increased release of all pro-inflammatory cytokines from C20 cells (eg, IL-2, IL-6, IL-8, IL-12, IFN-γ, TNF-α and IL-1β; Figures 6 and 7). For microglia treated with TNF-α (Figure 6), IL-6 and IL-8 were the cytokines with the highest concentrations present in the conditioned media. The mean values registered for IL-6 after TNF-α stimulation were ~172-fold higher than the untreated control. IL-8 exhibited an ~72.6-fold increase in TNF-α-treated samples versus untreated cells. A more modest, yet significant up-regulation in cytokines levels was observed for IL-2, IL-12, IL-1β and IFN-γ. IFN-γ levels were increased ~8-fold with treatment whilst the exposure of microglia to TNF-α produced increases varying from ~4.4-fold change for IL-2 to ~11.3-fold change for IL-1β. As for IL-4, IL-10 and IL-13, which are usually classified as anti-inflammatory cytokines, our data showed a similar trend where the concentration of these 3 cytokines was higher in immune-challenged samples compared to untreated microglia. For IL-4 and IL-10 the mean control values were ~0.02 and ~0.05 pg/ml, respectively. Following TNF-α treatment, IL-4 levels were increased ~14.5-fold while IL-10 levels increased ~82.4-fold. IL-13 levels in the media exhibited a ~38-fold increase when cells were exposed to TNF-α.

The impact of the glucocorticoid DEXA on TNF-α-mediated cytokine release was assessed by pre-treating microglial cells with DEXA for 1 hour before the cytokine challenge. This revealed that the addition of DEXA was sufficient to decrease the concentration of most of the examined cytokines in response to TNF-α. For proteins belonging to the pro-inflammatory group, it was observed that IL-6, IL-8 and IL-1β levels were negatively influenced by the action of the GC with a reduction of ~45% for IL-8 and ~86% for IL-6 compared to TNF-α alone. As for IL-1β, although the concentration of this cytokine in media from TNF-α-treated cells was already low, the pre-treatment of C20 cells with DEXA produced a significant ~28% down-regulation of IL-1β in the conditioned media compared to TNF-α treatment alone. A similar effect was also measured for IL-4 in which levels reached ~0.08 from ~0.29 pg/ml after pre-treatment with DEXA as well as in IL-10 in which we observed a decrease from ~4.12 to ~1.46 pg/ml. Pre-treatment of C20 cells with DEXA did not significantly alter levels of IFN-γ, IL-2, IL-12 and IL-13 in the conditioned media compared to TNF-α treatment alone.

IL-1β stimulation of C20 cells resulted in increased concentrations of all cytokines examined in the conditioned media (Figure 7). Interestingly, it was observed that similarly to treatment with TNF-α, IL-6 and IL-8 exhibited the highest up-regulation compared to the untreated control samples. In untreated C20 cell conditioned media the mean concentration value for IL-6 was ~2.0 pg/ml whilst in IL-1β-treated cells this value reached ~1157 pg/ml, a ~579-fold increase. Similarly, IL-8 levels increased ~94-fold in IL-1β challenged microglia. Additionally, there were significant increases in the levels of IFN-γ (~1258 vs ~0.57 pg/ml) and IL-4 (~147 vs ~0.02 pg/ml) in stimulated C20 cells. For the remaining cytokines analysed (TNF-α, IL-2, IL-10, IL-12 and IL-13) the values in the control group ranged from roughly 0.05 pg/ml (measured in IL-10) to 4.3 pg/ml (measured in IL-13). After exposure to IL-1β, microglia demonstrated an increase ranging from ~4.27-fold change (in IL-13) to 41.5-fold change (in IL-12). With the sole exception of IL-8, the levels of all the other analysed cytokines were dampened in cells pre-treated with DEXA compared to those stimulated with IL-1β alone. Indeed, there was a ~43% to ~82% reduction in the concentration of the following cytokines following DEXA treatment: TNF-α (51.4%), IL-2 (60%), IL-6 (82.3%), IL-13 (70%) and IL-10 (43%). A larger effect was measured for IL-12 and IFN-γ, where the concentration of these cytokines was reduced ~67% compared to treatment with IL-1β alone. Levels of IL-12 in the media were ~1.98 pg/ml after the cell exposure to DEXA compared to ~6.23 pg/ml in IL-1β sole stimulated samples, whilst for IFN-γ levels in the media were ~1258 compared to ~421 pg/ml in DEXA pre-treated samples. Notably, the greatest effect was measured for IL-4 with its concentration dropping from ~147 to ~1.04 pg/ml following DEXA pre-treatment in IL-1β stimulated cells.

For almost all cytokines analysed the cells treated with IL-1β exhibited the highest concentration of cytokines released into the medium. Overall, these results indicate that *in vitro* C20 microglial cells can exhibit a mixed activated phenotype with the increased production of both pro-inflammatory cytokines (eg, IL-6 and IL-8) and anti-inflammatory cytokines (eg, IL-4 and IL-13).

### TNF-α Treatment Modulates the Expression of Genes Involved in Inflammatory Responses and Negatively Impacts Neurodevelopmental Processes in Microglia

We next sought to explore transcriptional changes occurring within the C20 microglia. We employed RNA-sequencing to identify the possible presence of differentially expressed genes (DEGs) linked to distinctive experimental conditions that might be specifically involved in the cellular processes previously described.

In TNF-α-stimulated C20 cells, 2100 genes were found to be differentially expressed compared to the untreated control. Among these, we found a total of 1452 genes which were up-regulated. GO-term enrichment analysis confirmed the positive activation of the C20 cells, revealing that most of the examined transcripts were involved in biological processes characteristic of activated microglia such as those pathways implicated in the defence against microorganisms, cytokine-mediated signalling and in the initiation of the inflammatory response (Table 2). As expected, genes activated by the TNF-α signalling pathway were found to be enriched, such as *BIRC2* and *BIRC3*, which modulate inflammatory signalling and immunity; *ICAM1* and *VCAM1* which are involved in the process of cell adhesion and migration; *IL18R1* which is responsible for the binding of the proinflammatory cytokine IL-18; and several chemokines such as *CCL2*, *CCL20*, *CXCL6*, *CXCL3*, *CXCL1*.

**Table 2.** Gene Ontology and Protein Pathways Enriched for Up-Regulated DEGs in TNF-α Stimulated Microglia Versus Untreated Control Cells.

*Biological process (Gene Ontology)*			
*GO term*	*Description*	*Gene count*	*False discovery rate*
GO:0034097	Response to cytokine	79	2.07E-22
GO:0006952	Defence response	83	5.52E-21
GO:0019221	Cytokine-mediated signalling pathway	58	1.50E-19
GO:0006955	Immune response	86	6.63E-18
GO:0051607	Defence response to virus	33	3.29E-17
GO:0009605	Response to external stimulus	100	4.54E-15
GO:0044419	Interspecies interaction between organisms	89	4.92E-15
*KEGG pathway*			
*Pathway*	*Description*	*Gene count*	*False discovery rate*
hsa04668	TNF signalling pathway	21	3.40E-12
hsa05164	Influenza A	17	2.59E-06
hsa04064	NF-kappa B signalling pathway	13	8.34E-06
hsa04060	Cytokine-cytokine receptor interaction	18	0.00031
hsa04621	NOD-like receptor signalling pathway	14	0.00031
hsa05202	Transcriptional misregulation in cancer	14	0.00031
hsa04657	IL-17 signalling pathway	10	0.00046
*UniProt keywords*			
*Pathway*	*Description*	*Gene count*	*False discovery rate*
KW-0051	Antiviral defence	28	4.91E-18
KW-0391	Immunity	39	3.10E-11
KW-0399	Innate immunity	28	3.36E-09
KW-0395	Inflammatory response	17	2.03E-06
KW-0145	Chemotaxis	10	0.0017
KW-0202	Cytokine	13	0.0044

In addition, we detected in our cytokine-treated samples a cluster of genes (obtained following STRING analysis) that regulates the NF-kB pathway, which is activated by TNF-α-signalling and is responsible for immune response initiation. Another significant process that emerged as enriched from this analysis was the NOD-like receptor signalling pathway, which specialises in recognising non-self-components within the body. Furthermore, in these cells, chemokine or cytokine-mediated cellular responses, including the activation of the IL-17 pathway, were significantly up-regulated, suggesting the positive initiation of the inflammatory response as well as the chemotaxis of in vitro microglial cells.

In support of the qPCR results obtained regarding gene expression of the KP enzymes, increases in both *IDO-1* (6.3-fold change) and *KMO* (3.5-fold change) were also detected in TNF-α stimulated C20 using RNA-seq.

The number of down-regulated genes identified in TNF-α-treated C20 compared to control samples was 648 in total. In particular, most enriched pathways observed were involved in different development processes (Table 3). Notably, it was shown that the treatment of microglial cells with TNF-α had a considerable effect on genes participating in nervous system development and in the process of neurogenesis with a down-regulation of 82 and 63 of transcripts, respectively.

**Table 3.** Gene Ontology and Protein Domain Tables for Down-Regulated DEGs in TNF-α Versus Untreated Microglia.

*Biological process (Gene Ontology)*			
*GO term*	*Description*	*Gene count*	*False discovery rate*
GO:0007275	Multicellular organism development	140	0.00072
GO:0007399	Nervous system development	82	0.00072
GO:0022008	Neurogenesis	63	0.00072
GO:0048731	System development	127	0.00072
GO:0048856	Anatomical structure development	146	0.0011
GO:0065009	Regulation of molecular function	136	0.0011
GO:0032502	Developmental process	154	0.0017
GO:0030154	Cell differentiation	107	0.0034
GO:0048869	Cellular developmental process	108	0.0035
GO:0060284	Regulation of cell development	40	0.0043
GO:0050794	Regulation of cellular process	249	0.0045
*UniProt keywords*			
*Pathway*	*Description*	*Gene count*	*False discovery rate*
KW-0025	Alternative splicing	246	7.79E-06
KW-0965	Cell junction	35	0.0021
KW-0597	Phosphoprotein	189	0.0164
KW-0770	Synapse	22	0.0164
KW-0863	Zinc-finger	55	0.0164
KW-0539	Nucleus	130	0.0203
KW-0805	Transcription regulation	65	0.0427

### Treatment of Microglial Cells With DEXA and TNF-α Resulted in the Down-Regulation of Genes Involved in Cancer and Regulation of Cell Signalling

We then compared DEGs from cells pre-treated with DEXA and then stimulated with TNF-α, with those obtained from samples solely stimulated with the pro-inflammatory cytokine. We found 533 down-regulated genes and 416 up-regulated genes in cells that were pre-exposed to DEXA compared to cells treated with TNF-α alone. In the down-regulated genes, GO-term enrichment analysis showed enriched biological processes mainly involved in regulatory mechanisms such as response to stimulus, signal transduction and cellular communication (Table 4). Notably, KEGG-pathway enrichment analysis of the down-regulated genes, revealed that these genes are part of pathways related to cancers. More specifically, transcripts of the genes *TP53* and *TP63* were found to be down-regulated in TNF-α + DEXA-treated samples compared to samples treated with TNF-α alone. As for the KP enzymes, we observed a 6.3-fold increase in the expression of *IDO-1* in TNF-α versus control samples, but only a 3.2-fold increase in the expression of *IDO-1* in TNF-α + DEXA versus control samples, suggesting that there is a reduction of IDO-1 transcripts in cells pre-treated with DEXA.

**Table 4.** Gene Ontology and Protein Domain Tables for Down-Regulated DEGs in TNF-α Versus TNF-α + DEXA Microglia.

*Biological process (Gene Ontology)*			
*GO term*	*Description*	*Gene count*	*False discovery rate*
GO:0048518	Positive regulation of biological process	186	6.03E-09
GO:0048522	Positive regulation of cellular process	173	9.27E-09
GO:0048583	Regulation of response to stimulus	139	1.25E-08
GO:0010033	Response to organic substance	108	2.96E-07
GO:0010646	Regulation of cell communication	119	6.01E-07
GO:0007166	Cell surface receptor signalling pathway	89	7.13E-07
GO:0009966	Regulation of signal transduction	108	7.13E-07
GO:0022603	Regulation of anatomical structure morphogenesis	54	7.13E-07
GO:0023051	Regulation of signalling	119	7.13E-07
GO:0048584	Positive regulation of response to stimulus	87	7.13E-07
*KEGG pathway*			
*Pathway*	*Description*	*Gene count*	*False discovery rate*
hsa05205	Proteoglycans in cancer	18	5.04E-05
hsa05200	Pathways in cancer	25	0.0073
hsa04668	TNF signalling pathway	10	0.0125
hsa05202	Transcriptional misregulation in cancer	12	0.0165
hsa05224	Breast cancer	11	0.0165
*UniProt keywords*			
*Pathway*	*Description*	*Gene count*	*False discovery rate*
KW-0025	Alternative splicing	277	2.86E-14
KW-0964	Secreted	62	0.0038
KW-0732	Signal	94	0.0057
KW-0965	Cell junction	34	0.0057
KW-0325	Glycoprotein	117	0.0086
KW-0677	Repeat	124	0.0121
KW-0051	Antiviral defence	10	0.0279

On the other hand, it was observed that the up-regulated genes detected in the TNF-α + DEXA-stimulated samples were transcripts associated with enriched general cellular functions and predominantly regulatory processes associated with gene expression and cellular metabolism (Table 5). This was indeed confirmed by the results obtained from UniProt. Some significantly highlighted keywords were DNA-binding or zinc finger proteins, suggesting that the analysed enriched genes principally encoded proteins that function as transcription factors.

**Table 5.** Gene Ontology and Protein Domain Tables for Up-Regulated DEGs in TNF-α Versus TNF-α + DEXA Microglia.

*Biological process (Gene Ontology)*			
*GO term*	*Description*	*Gene count*	*False discovery rate*
GO:0006355	Regulation of transcription, DNA-templated	64	0.031
GO:0009889	Regulation of biosynthetic process	78	0.031
GO:0010468	Regulation of gene expression	86	0.031
GO:0019219	Regulation of nucleobase-containing compound metabolic process	74	0.031
GO:0031323	Regulation of cellular metabolic process	105	0.031
GO:0048523	Negative regulation of cellular process	84	0.031
GO:0050794	Regulation of cellular process	157	0.031
GO:0051252	Regulation of RNA metabolic process	69	0.031
GO:0065009	Regulation of molecular function	86	0.031
GO:0080090	Regulation of primary metabolic process	103	0.031
GO:0060255	Regulation of macromolecule metabolic process	103	0.0358
*UniProt keywords*			
*Keyword ID*	*Description*	*Gene count*	*False discovery rate*
KW-0025	Alternative splicing	162	0.14
KW-0597	Phosphoprotein	133	0.16
KW-0863	Zinc-finger	46	0.36
KW-0805	Transcription regulation	52	0.3
KW-0539	Nucleus	89	0.17
KW-0238	DNA-binding	41	0.26

## Discussion

Microglia are the first line of defence of the CNS and therefore have a prominent role in maintaining homeostasis within the brain. In the case of an unbalanced immune response due to constantly activated microglial cells, the release of neurotoxic factors results in a state of continuous inflammation which can cause neuronal dysfunction and ultimately lead to cellular death.^34^ Additionally, specific enzymes of the KP are expressed in microglial cells, and these have been identified to have a significant impact on pathological processes within the CNS. Specifically, the increased activity of the enzyme KMO during the immune response contributes to the production of the neurotoxic KP metabolites 3-HK and QUIN that can cause excitotoxicity and exacerbate neuroinflammation. Considering that KMO function in microglia has not been fully elucidated yet and that immune response alterations in these cells have been identified as important players in many diseases affecting the CNS, the aim of this study was to assess the downstream effect of various immune cytokines or LPS challenges on microglia cellular responses, such as the production of cytokines, together with a specific focus on the activation of the KP as determined by the expression of key KP enzymes. In addition, microglial cells were pre-treated with DEXA to test if the use of GC could modify microglial immune responses.

The C20 human microglial cell line was initially exposed to different immune stimulations (IL-1β, TNF-α, IFN-γ or LPS), alone or combined, to quantify the mRNA levels of *KMO* and *IDO-1*. As expected, in untreated control samples, the expression of both genes was very low. From our results it appears that the 2 pro-inflammatory cytokines, IFN-γ and TNF-α, have varying effects on the regulation of the expression of *IDO-1* and *KMO*, indicating that the mechanisms governing gene regulation for these enzymes might differ, even when they are stimulated by the same cytokines. The expression of *IDO-1* is significantly influenced by IFN-γ, however the effect of IFN-γ on *KMO* gene expression is not as pronounced. In contrast, TNF-α seems to have a more balanced impact affecting both enzymes more equally. The observation that IFN-γ and TNF-α have distinct effects on the expression of *IDO-1* and *KMO* suggests that there are likely different regulatory mechanisms at play for these enzymes. Even though the same pro-inflammatory cytokines are involved, the way they interact with the genes and the molecular pathways involved in their expression might vary. This could be due to differences in the specific receptors, signalling pathways and transcription factors that are engaged by IFN-γ and TNF-α leading to differential expression of these enzymes. In regard to *KMO* expression, with the sole exception of samples treated with IFN-γ, a significant up-regulation of the gene transcripts was observed in all the other immune stimulations, reaching a 50-fold change in TNF-α-treated cells, which was the highest *KMO* expression observed. This result is a novel finding in human microglial cells as, to date, the correlation between TNF-α and *KMO* expression has only been described regarding the parallel up-regulation of both this cytokine and the KP enzyme following other pro-inflammatory stimulations.^46^ DNA promoter regions of both *KMO* and *IDO-1* contain sequences predicted to be bound by NF-κB (www.genecards.org), which, in turn, controls the expression of genes implicated in the immune response and cell survival.^47^ As TNF-α stimulation is directly connected to the action of the NF-κB pathway, it is plausible to hypothesise that the observed increase of *KMO* expression could depend on the activation of NF-κB, which then translocates to the nucleus and binds to *KMO* specific DNA sequences regulating its transcription.

Although Heyes et al^48^ reported that IFN-γ produced an increase in KMO activity in monocytes, our results are in agreement with other studies in which low levels of *KMO* transcripts were observed in primary human neurons^49^ and human skin fibroblasts^50^ after stimulation with IFN-γ. Interestingly, the same treatment produced a completely different outcome in relation to *IDO-1* gene expression. It has been shown previously that IFN-γ treatment can increase *IDO-1* expression,^10^ and therefore we used this cytokine as a positive control and compared the effect of TNF-α immune challenge to investigate the expression of *IDO-1* in C20 cells. As predicted, *IDO-1* expression was dramatically increased in cells stimulated with IFN-γ as well as TNF-α. In the promoter region of the *IDO-1* gene, but not *KMO*, there are IFN-stimulated response elements (ISRE) which are necessary to activate the gene transcription initiated by IFN-γ.^51^ On the other hand, the TNF-α-activated NF-κB can influence the expression of *IDO-1* and *KMO*. Therefore, this could explain why only TNF-α, unlike IFN-γ, increased the transcription of both genes.

Subsequently, the effects of DEXA were investigated in C20 cells by adding the GC 1 hour before the immune stimulation. The quantification of *KMO* and *IDO-1* mRNA transcripts revealed that in cells exposed to DEXA there was a significant reduction in the expression of both genes when compared to the mRNA levels measured in samples solely challenged with TNF-α or IL-1β. As already mentioned, NF-κB can interact with *IDO-1* and *KMO* promoter regions. One of the known mechanisms of action used by natural GC to dampen inflammation induced by immune cells is to affect the transcription of genes activated by the NF-κB pathway.^52^ In a study by Aghai et al^53^ the administration of DEXA to neonates with respiratory distress was followed by a suppressed expression of NF-κB in the cytoplasm and nuclei of the isolated mononuclear cells. It is therefore reasonable to speculate that DEXA might indirectly regulate the activation of the KP through preventing NF-κB signalling. Our data also align with a previous study in which the treatment of the BV2 microglial cell line with DEXA prevented the up-regulation of *IDO-1* and *KMO* induced by IFN-γ.^54^ Another investigation in mice from Dostal et al^36^ showed that intraperitoneal injection of DEXA could ameliorate LPS-related inflammation in the periphery, however, researchers could measure an increase of a specific isoform of *IDO* by DEXA in glial cells. Similarly, the *IDO-1* transcript levels quantified from mice hippocampal slices were found to be up-regulated when DEXA was combined with IFN-γ, whilst *KMO* mRNA showed a significant reduction of the gene expression after DEXA administration.^55^ The contrasting results from in vitro and in vivo experimentation regarding the influence that DEXA exerts on *KMO* and *IDO-1* expression suggests that there might be a considerable discrepancy between measurements obtained from primary cells or tissues compared to cell lines, together with variations in the cellular response existing in different mammalian species exposed to the same stimulus.

The second part of this study was focussed on investigating the release of cytokines into the conditioned media of immune challenged microglia and examined whether the presence of DEXA had an impact on this feature of activated cells. Cytokines and chemokines are small proteins that act as messengers for intercellular communication and are involved in many biological processes including growth, apoptosis and activation. Depending on the role played during the immune response, these cytokines are commonly categorised as pro- or anti-inflammatory. The levels of 10 different cytokines were measured in conditioned media from C20 cells treated alone with TNF-α or IL-1β, or in combination with DEXA. We found that immune challenge of these microglia led to an increase in the production of several different cytokines. In particular, for both TNF-α or IL-1β stimulations the highest concentrations of cytokines in conditioned media compared to untreated cells were observed for IL-6 and IL-8, which are pro-inflammatory cytokines. It has been previously reported that following a peripheral immune challenge, microglia are the main source of IL-6 in the brain and that extended high concentrations of this cytokine plays a crucial pathogenic role in several neuroinflammatory diseases.^56^ IL-8 is a chemokine (also known as CXCL8), that is, produced by activated microglia in order to recruit other immune cells to the site of inflammation.^57^ The cytokine signature elicited in C20 cells recapitulates key aspects of the microglial component of acute neuroinflammation observed in vivo, particularly the robust induction of IL-1β, TNF-α, IL-6 and IL-8.^58-60^ However, the absence of multicellular interactions, blood-brain barrier influences and peripheral immune input in vitro results in narrower cytokine diversity and reduced resolution-phase complexity compared with whole-animal models.^58,59^

Notably, a significant increase in the levels of some anti-inflammatory cytokines (IL-4, IL-10 and IL-13) were also measured in the media. Indeed, IL-4 was increased following IL-1β treatment whilst the up-regulation of IL-13 levels was observed in cells stimulated with TNF-α. IL-10 levels were instead increased following both immune stimulations. Both C20 and in vivo models show induction of regulatory cytokines such as IL-10, although its onset is typically delayed in vivo as part of the resolution phase.^58,59^ This is likely due to the lack of clearance mechanisms, feedback loops and systemic immune regulation present in an intact organism.

It is known that inflammation is a natural response that helps to protect the organism against potential infections or injuries. Therefore, on one hand the release of pro-inflammatory molecules is necessary to attract immune cells and initiate the processes that can restore the homeostasis, whilst on the other hand the excessive production of these same factors can lead to tissue damage and chronic inflammation, further aggravating the condition. For this reason, the presence of anti-inflammatory mediators becomes necessary to control the dangerous effects of a prolonged immune response. One of the main functions of anti-inflammatory cytokines is to suppress the production of pro-inflammatory factors, such as IL-1β, TNF-α, prostaglandin E2 and iNOS in microglial cells,^61-63^ thus preventing excessive tissue damage. Moreover, IL-4 and IL-10 have also been reported to stimulate phagocytosis in a primary microglial culture,^64^ a vital feature that allows the removal of unwanted elements. Our data suggest that in vitro immune challenged microglia exhibit a mixed phenotype without unilaterally polarising towards the classic M1 state as expected when cells are exposed to a pro-inflammatory mediator.^65^ Similarly, it is usually accepted that the M2 alternative activation is mainly a prerogative of anti-inflammatory cytokine stimulation,^65^ however, in C20 cells both ‘neurotoxic’ and ‘neuroprotective’ cytokines were simultaneously induced by IL-1β and TNF-α treatments. This is consistent with recent advances, particularly employing single-cell transcriptomics, that have revealed how such a rigid dichotomy does not accurately capture the broad spectrum of microglial phenotypes and functions in the brain which often co-express both M1 and M2 markers.^66^

The addition of DEXA prior to the treatment of C20 with TNF-α and IL-1β was tested to ascertain any effect this synthetic GC might have on immune responses in this microglial cell line and thus confirm and extend its already established anti-inflammatory properties.^35-37^ The results obtained from our study showed that pre-treatment of C20 cells with DEXA is sufficient to reduce the levels of cytokines released into the media. Specifically, DEXA treatment prior to TNF-α or IL-1β stimulation resulted in a considerable down-regulation of pro-inflammatory cytokine release for IL-6, IL-8, IL-2, IL-12, TNF-α and IFN-γ compared to cytokine levels released from cells treated with TNF-α or IL-1β alone. Notably, this also applied to IL-4, IL-10 and IL-13 which are anti-inflammatory cytokines. Given that NF-κB regulates the expression of pro- and anti-inflammatory proteins involved in the immune response^67^ activated by TNF-α or IL-1β stimulation,^68^ DEXA may act by disrupting NF-κB immune-activation and thereby indirectly reducing the expression of cytokine genes.

Interestingly, our data also revealed that there is a less prominent effect of DEXA in IL-1β-treated cells compared to samples stimulated with TNF-α. Indeed, IL-8 concentrations were reduced in the media only when microglia were exposed to DEXA combined with TNF-α, whilst IL-6 showed a ~7.4-fold decrease in TNF-α and only a ~1.2-fold decrease in IL-1β-treated cells. These results might suggest that the strength of the anti-inflammatory properties of DEXA in microglial cells in vitro could depend on the type of the immune stimulation, and in particular, it seems that the impact of DEXA becomes more efficient when the cellular response derives from TNF-α stimulation instead of IL-1β. This phenomenon may be due to differential kinetics controlling the activation and the deactivation of NF-κB, which depends on the type of cytokine involved as has been described previously.^68^ Furthermore, there may be additional pathways activated by these cytokines that contribute to the transcription of the genes involved in the immune response. It is therefore plausible that DEXA has a stronger effect on TNF-α dependent cellular responses because IL-1β treatment more robustly stimulates the NF-κB pathway.

The use of RNA-seq in combination with GO-term enrichment analysis and KEGG-pathway enrichment analysis, was employed to compare the transcriptomic profiles of C20 cells treated with TNF-α with and without pre-treatment with DEXA, and to establish enrichment of molecular pathways connected to the microglial immune response. We sought to achieve a broader systems-level understanding of treatment-induced perturbations, in addition to the candidate KP genes, to capture broader pathway alterations. Compared to unstimulated control samples, genes upregulated in C20 cells treated with TNF-α showed an enrichment in pathways related to immunity and defence such as that of the NF-κB signalling pathway or NOD-like receptors, and therefore positively correlated microglia physiological reactivity to pro-inflammatory stimuli. This is not surprising, considering that the production of mediators for intercellular communication, such as cytokines and chemokines, are regulated through the NF-κB pathway.^67^ Another essential element of the immune response is the signalling pathway specifically activated by the NOD-like receptors. These highly specialised sensors are indeed able to recognise non-self-components, playing a fundamental role in defending the organism against invading microbes.^69^ In addition, when switched on, this pathway prompts the increase in IL-1β production as well as the expression of other NF-κB-regulated genes.^69^ Furthermore, we also observed that exposure to TNF-α generated a positive up-regulation of genes associated with IL-17-signalling which, in turn, can enhance the release of cytokines and chemokines together with neurotrophic factors.^70^ This suggests a combined pro- and anti-inflammatory effect of TNF-α on microglia and supports the data obtained from the analysis of the conditioned media in which we measured the production of both pro- and anti-inflammatory cytokines in TNF-α-treated cells. Consistent with our qPCR gene expression results, the RNA-seq transcriptomics data showed a significant increase in *IDO-1* and *KMO* gene expression levels following cellular exposure to TNF-α, confirming the key role of neuroinflammation on KP activation.

In contrast, 648 genes encoding for proteins involved in processes connected to nervous system development and neurogenesis were found to be down-regulated in TNF-α -treated C20 cells. There are several studies that have demonstrated how TNF-α can have a negative impact on neuronal differentiation. In 2011, Peng et al^71^ reported how culturing human foetal neural progenitor cells (NPC) with TNF-α-conditioned medium inhibited NPC neurogenesis. In a similar manner, the decrease in NPCs differentiating into neurons after exposure to media from immune-stimulated microglia was prevented by the addition of a TNF-α inhibitor to the culture media.^72^ Additionally, TNF-α released from activated BV2 microglial cells was observed to have a negative impact on both the viability and differentiation of dopaminergic neuron precursors.^73^ Our findings are in agreement with these studies, highlighting the importance of microglia in creating a correct neuronal network during development, as well as maintaining into adulthood a functioning and healthy brain. Moreover, this also indicates that prolonged exposure of the CNS to a pro-inflammatory environment may be severely detrimental to the process of neurogenesis or neural differentiation.

To the best of our knowledge, this is the first study to use transcriptomic analysis to identify molecular pathways that are influenced by the action of DEXA pre-treatment on immune-stimulated human microglial cells. For this reason, we also chose to compare DEGs in TNF-α + DEXA-treated cells to samples solely stimulated with TNF-α. Our analysis showed the presence of 533 down-regulated genes and 416 up-regulated genes in cells that were pre-exposed to DEXA followed by TNF-α stimulation compared to cells treated with TNF-α alone. In the first group, we found transcripts involved in mechanisms of cellular communication, such as response to stimulus or signal transduction. This indicates that the addition of DEXA influenced the action of TNF-α on microglia by reducing the response of the cells to the pro-inflammatory stimulus. As previously stated, one of the anti-inflammatory properties of DEXA was established to be the disruption of NF-κB immune-activated genes,^52^ which was also showed by our aforementioned data in which cells co-treated with TNF-α and DEXA presented a reduction in levels of various cytokines in the culture media. This effect was also seen in significantly enriched pathways related to cancers. STRING protein-protein association network analysis revealed that TP53 and TP63 transcripts were down-regulated in DEXA-treated samples compared to TNF-α only. These are tumour-suppressor proteins whose role is regulating the expression of genes involved in cell cycle, apoptosis, cancer cell migration or senescence.^74^ More specifically, it was reported by Aloi et al^75^ that the activation of TP53 supports the expression of microRNAs that modulate microglia pro-inflammatory responses whilst suppressing tissue repair and anti-inflammatory functions. Moreover, we also observed that although being present in the up-regulated group, *IDO-1* expression in TNF-α + DEXA samples was lower by ~50% compared to microglial cells solely treated with TNF-α. Overall, our data suggest a beneficial role of DEXA administration regarding dampening of inflammation as well as reducing the activation of the KP in pro-inflammatory stimulated microglia. As for the up-regulated genes, GO-term analysis showed that the enriched genes observed were mainly transcription factors associated with general cellular functions and regulatory processes of gene expression and metabolism, indicating that DEXA treatment can indeed produce beneficial effects on the inflammatory response without negatively affecting microglial physiology.

## Conclusion

Our study provides new evidence regarding important mechanisms activated in human microglial cells by immune challenges and further characterises the C20 microglial cell line as a robust and physiologically relevant human microglial model for exploring human-specific microglial biology and function.

More specifically, we have shown the up-regulation of *IDO-1* and *KMO* mRNA levels in response to a range of inflammatory cytokines, as well as the release of pro- and anti-inflammatory cytokines. All of these effects were significantly dampened by the addition of DEXA. In addition, analysis of the transcriptome also supported and extended the data obtained by highlighting enriched pathways activated by exposure to TNF-α and those downregulated in samples pre-treated with DEXA, emphasising the crucial role of synthetic GCs in moderating the microglial immune response induced in vitro by pro-inflammatory signals.
